# Comparison by Race of Conservative Management for Low-Risk and Intermediate-Risk Prostate Cancers in Veterans From 2004 to 2018

**DOI:** 10.1001/jamanetworkopen.2020.18318

**Published:** 2020-09-28

**Authors:** Ravi B. Parikh, Kyle W. Robinson, Sumedha Chhatre, Elina Medvedeva, John P. Cashy, Shika Veera, Joshua M. Bauml, Tito Fojo, Amol S. Navathe, S. Bruce Malkowicz, Ronac Mamtani, Ravishankar Jayadevappa

**Affiliations:** 1 Corporal Michael J. Crescenz VA Medical Center, Philadelphia, Pennsylvania; 2 Center for Health Equity Research and Promotion, Philadelphia, Pennsylvania; 3 Perelman School of Medicine, University of Pennsylvania, Philadelphia; 4 Abramson Cancer Center, University of Pennsylvania, Philadelphia; 5 Leonard Davis Institute of Health Economics, University of Pennsylvania, Philadelphia; 6 Herbert Irving Comprehensive Cancer Center, the College of Physicians and Surgeons at Columbia University, New York, New York

## Abstract

**Importance:**

Conservative management (ie, active surveillance or watchful waiting) is a guideline-based strategy for men with low-risk and intermediate-risk prostate cancer. However, use of conservative management is controversial for African American patients, who have worse prostate cancer outcomes compared with White patients.

**Objective:**

To examine the association of African American race with the receipt and duration of conservative management in the Veterans Health Administration (VA), a large equal-access health system.

**Design, Setting, and Participants:**

This cohort study used data from the VA Corporate Data Warehouse for 51 543 African American and non-Hispanic White veterans diagnosed with low-risk and intermediate-risk localized node-negative prostate cancer between January 1, 2004, and December 31, 2013. Men who did not receive continuous VA care were excluded. Data were analyzed from February 1 to June 30, 2020.

**Exposures:**

All patients received either definitive therapy (ie, prostatectomy, radiation, androgen deprivation therapy) or conservative management (ie, active surveillance or watchful waiting).

**Main Outcomes and Measures:**

Receipt of conservative management and (for patients receiving conservative management) time from diagnosis to definitive therapy.

**Results:**

The median (interquartile range) age of the 51 543 veterans in our cohort was 65 (61-70) years, and 14 830 veterans (28.8%) were African American individuals. Compared with White veterans, African American veterans were more likely to have intermediate-risk disease (18 988 [51.7%] vs 8526 [57.5%]), 3 or more comorbidities (15 438 [42.1%] vs 7614 [51.3%]), and high disability-related or income-related needs (9078 [24.7%] vs 4614 [31.1%]). Overall, 20 606 veterans (40.0%) received conservative management. African American veterans with low-risk disease (adjusted relative risk, 0.95; 95% CI, 0.92-0.98; *P* < .001) and intermediate-risk disease (adjusted relative risk, 0.92; 95% CI, 0.87-0.97; *P* = .002) were less likely to receive conservative management than White veterans. Compared with White veterans, African American veterans with low-risk disease (adjusted hazard ratio, 1.71; 95% CI, 1.50-1.95; *P* < .001) and intermediate-risk disease (adjusted hazard ratio, 1.46; 95% CI, 1.27-1.69; *P* < .001) who received conservative management were more likely to receive definitive therapy within 5 years of diagnosis (restricted mean survival time [SE] at 5 years, 1679 [5.3] days vs 1740 [2.4] days; *P* < .001).

**Conclusions and Relevance:**

In this study, conservative management was less commonly used and less durable for African American veterans than for White veterans. Prospective trials should assess the comparative effectiveness of conservative management in African American men with prostate cancer.

## Key Points

**Question:**

Are there racial differences in receipt and duration of conservative management (ie, active surveillance or watchful waiting) among veterans with low-risk and intermediate-risk prostate cancer?

**Findings:**

In this cohort study of 51 543 veterans with low-risk and intermediate-risk prostate cancer, African American veterans were less likely than White veterans to receive conservative management, and African American patients who received conservative management were more likely to receive definitive therapy within 5 years of diagnosis.

**Meaning:**

The findings of this study suggest that conservative management for low-risk and intermediate-risk prostate cancer may be less durable for African American veterans compared with White veterans.

## Teaser

This cohort study examines the association of African American race with the receipt and duration of conservative management (ie, active surveillance or watchful waiting) in the Veterans Health Administration from 2004 to 2018.

## Introduction

Conservative management of low-risk or intermediate-risk prostate cancer with active surveillance or watchful waiting is a guideline-based alternative to definitive prostatectomy or radiation.^1^ In light of evidence suggesting that conservative management may delay the toxic effects of definitive therapy without compromising outcomes in low-risk and intermediate-risk disease, rates of conservative management have increased substantially during the past decade.^2,3^

However, seminal clinical trials of conservative management had a low enrollment of African American men.^4,5,6^ Additionally, among Medicare beneficiaries with low-risk prostate cancer, African American men may have higher mortality than non-Hispanic White men, even after controlling for socioeconomic status.^7^ This raises the possibility of race-based, biological differences in low-risk prostate cancer and has led to a debate regarding whether African American patients should be candidates for conservative management.^8,9,10^ Understanding racial differences in the uptake and duration of conservative management is key to counseling African American men regarding the risks and benefits of conservative management.

We examined differences in the receipt of conservative management between African American and White veterans with low-risk and intermediate-risk localized prostate cancer who received care within the Veterans Health Administration (VA). Among men who received conservative management, we also examined race-based differences in time to definitive therapy, a metric assessing the duration and effectiveness of conservative management.^11,12^ We hypothesized that receipt of conservative management and time to definitive therapy would be similar between African American and White veterans receiving care within an equal-access health system.^13^

## Methods

This study followed the Strengthening the Reporting of Observational Studies in Epidemiology (STROBE) reporting guideline.^14^ This study was approved by the Corporal Michael J. Crescenz VA Medical Center institutional review board with a waiver of informed consent owing to the use of deidentified data.

### Data Sources

Administrative health care data from January 1, 2004, to December 31, 2018, were obtained from the VA Corporate Data Warehouse (CDW), a national database housing inpatient, outpatient, laboratory, procedure, and pharmacy encounters throughout the VA as well as fee-basis care received in non-VA facilities and covered by the VA.^15,16^ We obtained data from patients with biopsy-proven or clinically suspected prostate cancer from the VA CDW Oncology Raw registry, which contains direct extracts from CDW of oncology-specific data, including tumor markers and stage for patients with biopsy-proven or clinically suspected cancer.

### Cohort

We identified veterans as having prostate cancer if they had a diagnosis of prostate cancer in the registry and had at least 1 VA encounter with an *International Classification of Diseases, Ninth Revision, Clinical Modification* code for prostate cancer. We identified veterans with low-risk (prostate-specific antigen [PSA] level, <10 ng/mL [to convert to micrograms per liter, multiply by 1.0]; Gleason score, ≤6; stage cT1 or T2a at diagnosis) and intermediate-risk (PSA level, 10-20 ng/mL; Gleason score, 7; and stage T2b or T2c at diagnosis) localized prostate cancer who had a diagnosis date between January 1, 2004, and December 31, 2013, and who had no diagnosis code of prostate cancer in the year before diagnosis.^17^ Patients with missing clinical stage were excluded; patients with clinical stage TX disease were included if they met PSA and Gleason criteria for low-risk or intermediate-risk disease. As we were interested only in comparative associations between African American and White men, we excluded men whose race, defined by self-report and/or the CDW registry, was not classified as African American or non-Hispanic White. Patients who were younger than 21 years or had no structured inpatient or outpatient data were also excluded. Previous studies have suggested that White veterans are more likely to seek non-VA care than African American veterans.^18^ To approximate a cohort receiving VA-only care, we excluded patients with no recorded use of VA inpatient or outpatient health services in the year prior to diagnosis and who did not have documented follow-up with a VA primary care physician within each of the years that they were alive during the 5-year follow-up period. An illustration of our cohort selection is found in eFigure 1 in the Supplement.

### Outcomes

The coprimary outcomes were receipt of conservative management (in the full cohort) and time to definitive treatment (in the subcohort of patients who received conservative management). Receipt of conservative management was defined as the absence of definitive therapy (ie, prostatectomy, radiation, androgen deprivation therapy, or chemotherapy), as defined in the CDW registry and by procedure codes and pharmacy claims (eTable 1 in the Supplement), from diagnosis to 1 year after the date of diagnosis. This definition has been used in previous analyses within the VA.^2^ Patients classified as receiving conservative management were further categorized into active surveillance (ie, ≥2 PSA level tests and 1 biopsy within 2 years after diagnosis) or watchful waiting.^2^ Time to definitive therapy was defined as the time from the date of diagnosis to the date of definitive therapy, which was defined using dates from the registry or procedure codes. Follow-up was terminated at the earliest date of definitive therapy or death; for all others, follow-up was terminated 5 years after the diagnosis date.

### Covariates

Race (African American or White), as defined in the CDW, was the exposure of interest in all multivariable analyses. Other variables included in multivariable analyses were age at diagnosis, pre-treatment PSA level (continuous), time period of diagnosis, veteran enrollment priority group (a proxy for individual-level income-related and disability-related needs in the VA),^19^ marital status, Elixhauser comorbidity index,^20,21^ Area Deprivation Index,^22,23,24^ urban vs rural location of patient residence, and driving distance to the nearest tertiary VA medical center.

### Statistical Analysis

All data were extracted on December 31, 2019, and statistical analysis was conducted between February 1 to June 30, 2020. The distribution of demographic and clinical variables was summarized for all patients with low-risk and intermediate-risk prostate cancer and stratified by race. The distribution of variables was compared using χ^2^ tests for categorical variables, Cochran-Armitage test for ordinal variables, and *t* tests and Wilcoxon-Mann-Whitney tests for continuous variables.

To estimate the relative risk of receiving conservative management among patients with low-risk and intermediate-risk prostate cancer, we fit separate log-binomial regression modes for patients with low-risk and intermediate-risk prostate cancer at diagnosis, adjusted for all covariates, with the outcome being the receipt of conservative management. To estimate the risk of definitive treatment associated with race, we fit separate multivariable Cox proportional hazards regressions for patients with low-risk and intermediate-risk disease who received conservative management, adjusted for all covariates, with the outcome being receipt of definitive therapy. The proportional hazard assumption was tested with Kaplan-Meier curves, in which race was the categorical variable. In a secondary analysis, we calculated the difference in restricted mean survival time to receipt of definitive therapy at 5 years between African American and White veterans who received conservative management. In another secondary analysis estimating how the association between race and receipt of definitive treatment varies by conservative management strategy, we fit separate multivariable Cox proportional hazards models for patients receiving active surveillance vs watchful waiting. To better account for patients who initially elected conservative management but who progressed within 1 year of diagnosis and to minimize bias owing to inaccurate representation of the conservative management group, we performed a sensitivity analysis varying the definition of conservative management as the absence of definitive therapy from diagnosis to 6 months after the date of diagnosis.

Statistical analyses were performed using SAS Enterprise Guide version 7.15 (SAS Institute). A 2-sided *P* < .05 was considered statistically significant.

## Results

### Baseline Characteristics

Among 51 543 veterans, the median (interquartile range) age was 65 (61-70) years, 14 830 (28.8%) were African American individuals, 24 029 (46.6%) had a low-risk disease at the time of diagnosis, and 5109 (9.9%) died during the follow-up period (Table 1). The mean (SD) follow-up was 4.80 (0.72) years and did not differ between African American and White veterans. Compared with White veterans, African American veterans were more likely to have intermediate-risk disease (18 988 [51.7%] vs 8526 [57.5%]), have a high comorbidity burden (Elixhauser Comorbidity Index score ≥3: 15 438 [42.1%] vs 7614 [51.3%]), be unmarried (15 336 [41.8%] vs 8378 [56.5%]), and be in a high-needs enrollment priority group (9078 [24.7%] vs 4614 [31.1%]). African American veterans were more likely to receive radiation as primary treatment than White veterans (6101 [41.1%] vs 13 061 [35.6%]); this difference persisted across all time periods and risk groups (eTable 2 in the Supplement).

**Table 1.** Baseline Characteristics

Characteristics	Patients, No. (%)	
	African American (n = 14 830)	White (n = 36 713)
Follow-up, mean (SD), y	4.81 (0.71)	4.80 (0.73)
Age at diagnosis, median (IQR), y	63 (58-67)	65 (62-71)
Pretreatment PSA level, mean (SD), ng/mL	6.69 (3.64)	6.31 (3.42)
Area Deprivation Index, mean (SD)^a^	62.08 (21.7)	54.15 (20.9)
Driving time, mean, (SD), h	1.07 (1.4)	1.79 (2.1)
Risk group		
Low	6304 (42.5)	17 725 (48.3)
Intermediate	8526 (57.5)	18 988 (51.7)
Urban or rural residence		
Rural	3213 (21.7)	18 151 (49.4)
Urban	11 617 (78.3)	18 562 (50.6)
Marital status		
Married	6452 (43.5)	21 377 (58.2)
Single or divorced	8378 (56.5)	15 336 (41.8)
Enrollment priority		
1-2, Indicating highest need	4614 (31.1)	9078 (24.7)
3-4	1873 (12.6)	4324 (11.8)
5-6	6433 (43.4)	15 284 (41.6)
7-8, Indicating lowest need	1910 (12.9)	8027 (21.9)
Elixhauser Comorbidity Index score		
0	229 (1.5)	788 (2.1)
1-2	6987 (47.1)	20 487 (55.8)
≥3	7614 (51.3)	15 438 (42.1)
Initial management strategy		
Conservative management	5194 (35.0)	15 412 (42.0)
Active surveillance	784 (5.8)	13 061 (35.6)
Watchful waiting	2667 (19.5)	6379 (17.4)
Radiation	6101 (41.1)	13 061 (35.6)
Prostatectomy	2761 (18.6)	6379 (17.4)
Other	774 (5.2)	1861 (5.1)
Diagnosis date		
2004-2007	4811 (32.4)	13 178 (35.9)
2008-2011	6334 (42.7)	15 714 (42.8)
2012-2013	3685 (24.8)	7821 (21.3)
Died during follow-up	1425 (9.6)	3684 (10.0)

Abbreviation: IQR, interquartile range; PSA, prostate-specific antigen.

SI conversion factor: To convert PSA to micrograms per liter, multiply by 1.0.

^a^ The Area Deprivation Index allows for rankings of neighborhoods by socioeconomic status disadvantage at the national level. Higher Area Deprivation Index score corresponds to more disadvantaged neighborhoods.

### Receipt of Conservative Management

Overall, 20 606 veterans (40.0%) received conservative management; 12 069 of 24 029 veterans (50.2%) with low-risk and 8537 of 27 514 (31.0%) with high-risk localized prostate cancer received conservative management, respectively. During the study period, rates of receipt of conservative management in veterans with low-risk prostate cancer increased for both African American and White veterans but remained stable among those with intermediate-risk prostate cancer (eFigure 2 in the Supplement). After adjusting for all covariates, African American veterans with low-risk (adjusted relative risk, 0.95; 95% CI, 0.92-0.98; *P* < .001) and intermediate-risk (adjusted relative risk, 0.92; 95% CI, 0.87-0.97; *P* = .002) prostate cancer had a lower likelihood of receiving conservative management than White veterans. Higher age and higher comorbidity burden were associated with significantly higher likelihood of conservative management in univariable analyses of the low-risk and intermediate-risk cohorts (eTable 3 in the Supplement).

### Likelihood of Receiving Definitive Treatment Among Veterans Receiving Conservative Management

**Figure 1.**
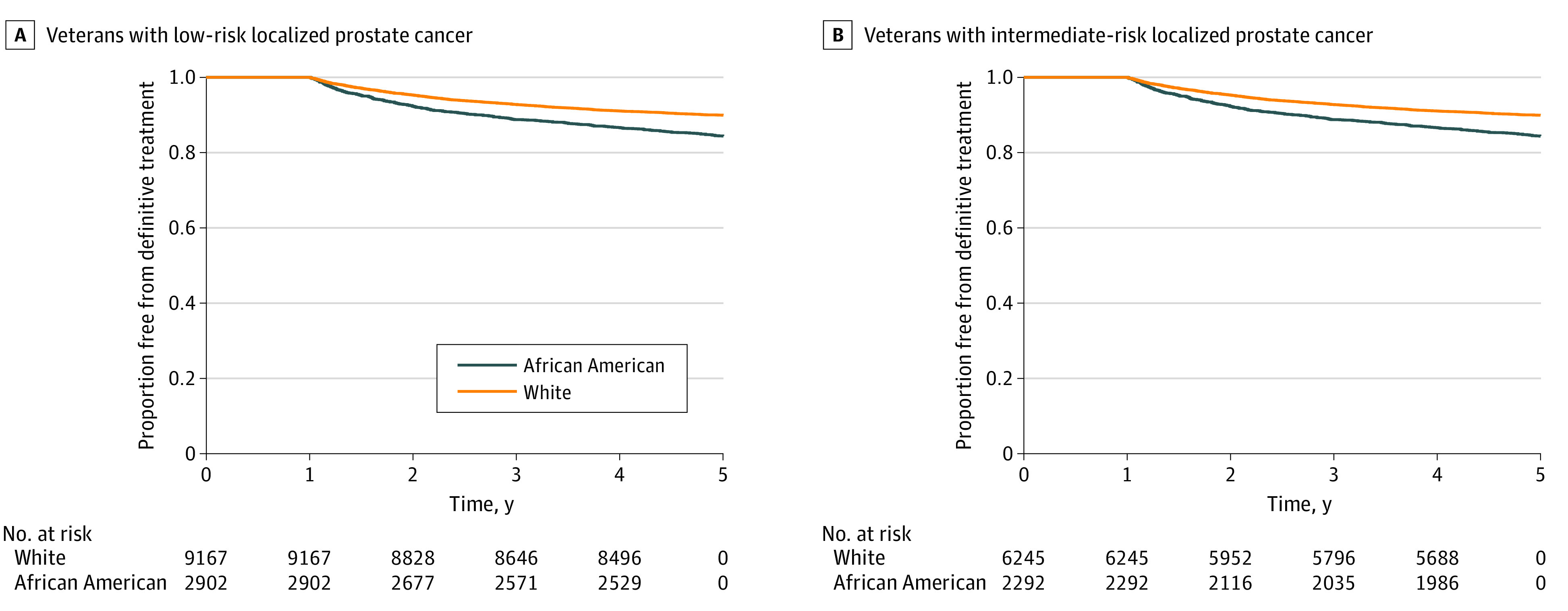
Time to Receipt of Definitive Therapy Among Veterans With Low-Risk and Intermediate-Risk Localized Prostate Cancer Who Received Conservative Management

Of 20 606 veterans who received conservative management, 2163 (10.5%) received definitive therapy during the 5-year follow-up. The median (interquartile range) time to definitive treatment was 719 (498-1085) days for African American men and 787 (524-1178) days for White men. The 5-year risk of receiving definitive therapy during the follow-up period was higher for African American veterans compared with White veterans (low-risk disease: adjusted hazard ratio [aHR], 1.71; 95% CI, 1.50-1.95; *P* < .001; intermediate-risk disease: aHR, 1.46; 95% CI, 1.27-1.69; *P* < .001) (Figure 1A and Figure 1B). Lower absolute PSA level, being married, and living in a rural area were associated with a lower risk of receiving definitive therapy within 5 years of diagnosis (Table 2). The restricted mean survival time (SE) at 5 years was significantly lower for African American veterans than White veterans (1679 [5.3] days vs 1740 [2.4] days; difference, 61 days; *P* < .001). Sensitivity analyses that used a shorter time window from diagnosis to define conservative management demonstrated similar results (eFigure 3A and eFigure 3B in the Supplement).

**Table 2.** Cox Proportional Hazard Multivariable Association of Likelihood of Definitive Therapy Among Veterans Receiving Conservative Management

Characteristic	Low-risk disease		Intermediate-risk disease	
	aHR (95% CI)	*P* value	aHR (95% CI)	*P* value
Race				
White	1 [Reference]	NA	1 [Reference]	NA
African American	1.71 (1.50-1.95)	<.001	1.46 (1.27-1.69)	<.001
Age at diagnosis	1.00 (0.99-1.01)	.57	1.01 (1.00-1.02)	.006
Absolute PSA level	1.09 (1.06-1.12)	<.001	1.05 (1.03-1.06)	<.001
Area Deprivation Index	1.00 (1.00-1.00)	.54	1.00 (1.00-1.01)	.09
Driving time	0.95 (0.91-1.00)	.03	1.02 (0.99-1.05)	.12
Urban/rural				
Urban	1 [Reference]	NA	1 [Reference]	NA
Rural	0.85 (0.75-0.98)	.02	0.78 (0.68-0.90)	<.001
Marital status				
Single or divorced	1 [Reference]	NA	1 [Reference]	NA
Married	0.78 (0.70-0.88)	<.001	0.79 (0.69-0.90)	<.001
Enrollment priority				
7-8	1 [Reference]	NA	1 [Reference]	NA
5-6	1.25 (1.07-1.47)	.006	1.29 (1.08-1.55)	.006
3-4	1.20 (0.90-1.38)	.30	1.25 (0.99-1.58)	.06
1-2	1.03 (0.86-1.23)	.77	1.25 (1.020-1.53)	.03
Elixhauser Comorbidity Index				
≥3	1 [Reference]	NA	1 [Reference]	NA
1-2	0.97 (0.86-1.09)	.62	0.99 (0.87-1.13)	.90

Abbreviations: aHR, adjusted hazard ratio; NA, not applicable; PSA, prostate-specific antigen.

### Mechanism of Conservative Management

**Figure 2.**
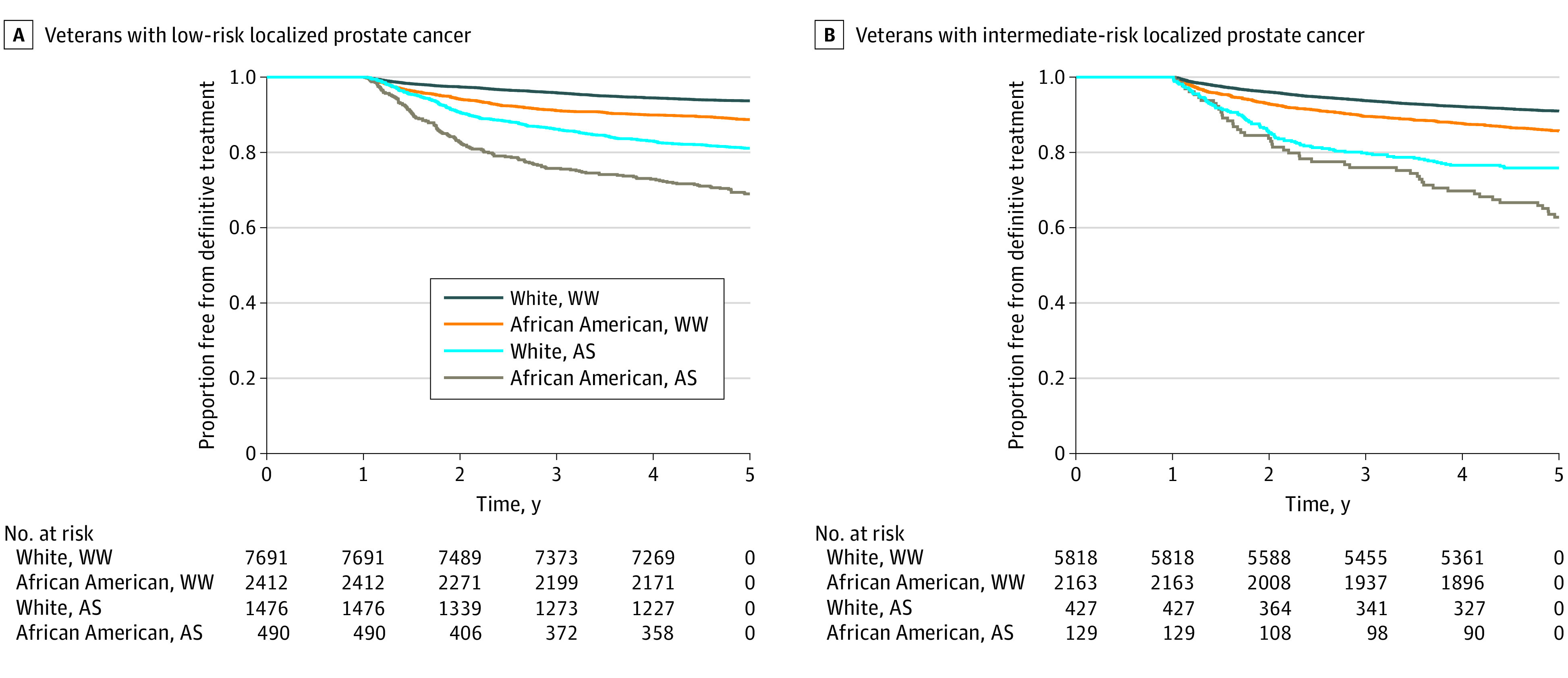
Time to Definitive Therapy Among Veterans With Low-Risk and Intermediate-Risk Localized Prostate Cancer Who Received Conservative Management, Stratified by Active Surveillance (AS) vs Watchful Waiting (WW)

Of 20 606 veterans who received conservative management, 2522 (12.2%) (1966 of 12 069 [16.3%] with low-risk disease and 556 of 8537 [6.5%] with intermediate-risk disease) received active surveillance. The proportion of veterans receiving active surveillance did not meaningfully differ between African American and White men with low-risk disease (490 of 2902 [16.9%] vs 1476 of 9167 [16.1%]) or intermediate-risk disease (129 of 2292 [5.6%] vs. 427 of 6245 [6.8%]). Among veterans receiving active surveillance, the likelihood of receiving definitive therapy was higher for African American patients compared with White patients (low-risk disease: aHR, 1.53; 95% CI, 1.23-1.90; *P* < .001; intermediate-risk disease: aHR, 1.56; 95% CI, 1.06-2.29; *P* = .03) (Figure 2A and Figure 2B). Among veterans receiving watchful waiting, the likelihood of receiving definitive therapy was also higher for African American patients compared with White patients (low-risk disease: aHR, 1.80; 95% CI, 1.53-2.12; *P* < .001; intermediate-risk disease: aHR, 1.48; 95% CI, 1.27-1.73; *P* < .001).

## Discussion

Using longitudinal national VA data, we demonstrated that African American veterans were slightly less likely to receive conservative management than White veterans (adjusted relative risk of 0.95 for patients with low-risk disease and of 0.92 for patients with intermediate-risk disease) with localized prostate cancer. Among patients receiving conservative management, African American veterans had a higher risk of receiving definitive therapy than White veterans (aHR of 1.71 for low-risk disease and 1.46 for intermediate-risk disease) within 5 years of diagnosis.

There are several strengths to this analysis. To our knowledge, this analysis is the largest to date to study the duration of conservative management in a large, equal-access health system and has a similar follow-up time compared with other population-wide analyses of conservative management, including those using a Medicare population.^25,26^ Such long follow-up is important given the natural history of localized prostate cancer.^27,28^ The nature of our data set, which contains both claims and electronic health record information from a large equal-access national population, is unique. Finally, the VA is an important setting to study racial differences in prostate cancer outcomes because African American representation is much higher in the veteran population than in the Medicare population.^29^ There are several findings worth emphasis.

First, our study is among the first to show that among men who receive conservative management, the time to definitive therapy is shorter in African American patients compared with White patients with low-risk and intermediate-risk prostate cancer. Our analysis controls for various socioeconomic confounders, including driving distance, area-level deprivation, and income and disability status, and studies a population within an equal-access health care system with a high representation of African American patients. This race-based difference persisted across different mechanisms of conservative management (ie, active surveillance or watchful waiting). Previous analyses have found an increased risk of mortality among African American men with low-risk prostate cancer, even when controlling for socioeconomic status.^7^ However, it has been difficult to disentangle the role that unmeasured nonclinical confounders (eg, access to care, patient-specific and group-specific care preferences) play compared with potential racial differences in the biology of low-risk prostate cancer.^30^

The mechanism behind the shorter duration of conservative management in African American men is unclear, given that we were unable to obtain PSA levels or pathology results prior to definitive therapy in the conservative management cohort. Recent studies have suggested that men of African descent may have an inherent biological susceptibility toward more aggressive low-risk and intermediate-risk prostate cancer,^31,32,33^ which could result in a faster need for definitive therapy. Indeed, we found that African American veterans who received active surveillance, which involves regular PSA checks and biopsies and may control for race-specific differences in follow-up, still had a faster time to definitive therapy. Alternatively, differences in treatment preferences, patient-level or clinician-level risk tolerance, or trust in the health system among African American veterans could also have contributed to this finding. Further qualitative and retrospective work among African American men receiving conservative management and their clinicians could elucidate these mechanisms. Racial differences should be further studied in the context of a prospective trial, given that they may warrant the consideration of differential management strategies for African American men with low-risk or intermediate-risk prostate cancer.

Second, our study adds to prior literature in both veteran and nonveteran populations suggesting that conservative management has become the predominant management strategy for men with low-risk prostate cancer but remains low for men with intermediate-risk prostate cancer.^2,3^ The rate of uptake of conservative management was similar to what has been reported in a previous analysis of conservative management trends in veterans with low-risk prostate cancer. This analysis found that African American race was associated with slightly higher odds of receipt of conservative management.^2^ In contrast, we found that African American patients had a slightly lower relative risk of receiving conservative management, although the low magnitude of this difference is unlikely to be clinically meaningful. The difference in these 2 VA-based studies may be because our cohort consisted of veterans who received consistent VA-based care, who may have different preferences regarding primary management strategies compared with veterans who seek non-VA care. Additionally, while prior studies have primarily examined trends in conservative management in patients with low-risk disease,^2,3,34^ we also examined trends in patients with intermediate-risk prostate cancer and found lower uptake of conservative management among African American patients. This is important given the increasing recognition that certain patients with intermediate-risk prostate cancer can receive conservative management with low long-term mortality risk.^35^ Our findings are consistent with prior research in Medicare populations, which also found lower uptake of active surveillance among African American patients, even when controlling for socioeconomic status.^36^ Lower rates of conservative management among African American men may be because of patient and/or clinician preference and should be explored through further qualitative work.

### Limitations

This study has limitations. First, our population was limited to veterans receiving primary treatment and active follow-up within the VA. While previous analyses have suggested that this may underestimate the degree of racial differences in laboratory testing,^37^ our data set did capture primary treatment and procedure codes from fee-basis care provided at non-VA hospitals and thus identified procedures (eg, radiation, surgery) done at non-VA hospitals that were paid through the VA. Furthermore, we attempted to control for receipt of non-VA care by identifying a cohort of veterans who primarily used VA care in all years of the follow-up period. Rates of receipt of conservative management in our study were nearly identical to rates described in a previously published analysis of veterans with prostate cancer that did link data to Medicare claims.^2^ Second, there may be systematic clinical differences between veterans and nonveterans owing to differential rates of screening and timing of detection of prostate cancer at the VA.^38,39^ Thus, our findings may not generalize to patients who receive their care primarily outside the VA. Third, we were unable to differentiate between unfavorable and favorable intermediate-risk prostate cancer in our data set. Conservative management is not a guideline-based management strategy in unfavorable intermediate-risk prostate cancer, and thus, it is possible that we have underestimated rates of receipt of guideline-based conservative management in the intermediate-risk cohort. However, findings regarding racial disparities were consistent between low-risk and intermediate-risk cohorts in our analysis, indicating that these disparities may exist in intermediate-risk disease as well. Fourth, our data set of routinely collected data did not contain information regarding prostate volume, which may also factor into decisions regarding pursuing active surveillance.^40^

## Conclusions

In this cohort study, we found that observed rates of conservative management increased at similar rates for African American and White veterans with low-risk disease but increased at much slower rates in veterans with intermediate-risk disease. After adjusting for demographic, diagnosis, tumor-specific, and socioeconomic covariates, African American patients were less likely to receive conservative management. Furthermore, the duration of conservative management was markedly lower for African American patients receiving either active surveillance or watchful waiting. Future prospective research should study the effectiveness of conservative management in African American men with low-risk and intermediate-risk prostate cancer to determine whether race-specific recommendations regarding conservative management are warranted.

## Notes

Supplement. **eFigure 1. **Cohort Selection **eFigure 2. **Observed Trends in Receipt of Conservative Management Among Veterans With Localized Prostate Cancer **eFigure 3. **Sensitivity Analysis of Time to Receipt of Definitive Therapy **eTable 1. **Code List **eTable 2. **Initial Management Strategy Over Time **eTable 3. **Univariable Log-Binomial Associations of Likelihood of Receipt of Conservative Management Across Patients With Low-Risk and Intermediate-Risk

## References

[1] Mohler JL, Horwitz EM, Richey S NCCN Guidelines Index table of contents discussion. Prostate Cancer. 2020;167:479-505.

[2] Loeb S, Byrne N, Makarov DV, Lepor H, Walter D Use of conservative management for low-risk prostate cancer in the Veterans Affairs Integrated Health Care System from 2005-2015. JAMA. 2018;319(21):2231-2233. doi:10.1001/jama.2018.5616 29800017 PMC6134433

[3] Mahal BA, Butler S, Franco I, Use of active surveillance or watchful waiting for low-risk prostate cancer and management trends across risk groups in the United States, 2010-2015. JAMA. 2019;321(7):704-706. doi:10.1001/jama.2018.19941 30743264 PMC6439610

[4] Hamdy FC, Donovan JL, Lane JA, ; ProtecT Study Group 10-Year outcomes after monitoring, surgery, or radiotherapy for localized prostate cancer. N Engl J Med. 2016;375(15):1415-1424. doi:10.1056/NEJMoa1606220 27626136

[5] Klotz L, Vesprini D, Sethukavalan P, Long-term follow-up of a large active surveillance cohort of patients with prostate cancer. J Clin Oncol. 2015;33(3):272-277. doi:10.1200/JCO.2014.55.1192 25512465

[6] Bill-Axelson A, Holmberg L, Garmo H, Radical prostatectomy or watchful waiting in prostate cancer–29-year follow-up. N Engl J Med. 2018;379(24):2319-2329. doi:10.1056/NEJMoa1807801 30575473

[7] Mahal BA, Berman RA, Taplin M-E, Huang FW Prostate cancer-specific mortality across Gleason scores in black vs nonblack men. JAMA. 2018;320(23):2479-2481. doi:10.1001/jama.2018.11716 30561471

[8] Silberstein JL, Feibus AH, Maddox MM, Active surveillance of prostate cancer in African American men. Urology. 2014;84(6):1255-1261. doi:10.1016/j.urology.2014.06.064 25283702

[9] Leinwand GZ, Gabrielson AT, Krane LS, Silberstein JL Rethinking active surveillance for prostate cancer in African American men. Transl Androl Urol. 2018;7(suppl 4):S397-S410. doi:10.21037/tau.2018.06.19 30363480 PMC6178310

[10] Schenk JM, Newcomb LF, Zheng Y, African American race is not associated with risk of reclassification during active surveillance: results from the Canary Prostate Cancer Active Surveillance Study. J Urol. 2020;203(4):727-733. doi:10.1097/JU.0000000000000621 31651227 PMC7384451

[11] Welty CJ, Cooperberg MR, Carroll PR Meaningful end points and outcomes in men on active surveillance for early-stage prostate cancer. Curr Opin Urol. 2014;24(3):288-292. doi:10.1097/MOU.0000000000000039 24614347 PMC6586410

[12] Olsson H, Nordström T, Clements M, Grönberg H, Lantz AW, Eklund M Intensity of active surveillance and transition to treatment in men with low-risk prostate cancer. Eur Urol Oncol. 2019;S2588-9311(19)30072-0. doi:10.1016/j.euo.2019.05.005 31235395

[13] Dess RT, Hartman HE, Mahal BA, Association of black race with prostate cancer-specific and other-cause mortality. JAMA Oncol. 2019;5(7):975-983. doi:10.1001/jamaoncol.2019.0826 31120534 PMC6547116

[14] The EQUATOR Network. The Strengthening the Reporting of Observational Studies in Epidemiology (STROBE) Statement: guidelines for reporting observational studies. Accessed November 14, 2019. https://www.equator-network.org/reporting-guidelines/strobe/

[15] US Department of Veterans Affairs Corporate Data Warehouse (CDW). Accessed November 14, 2019. https://www.hsrd.research.va.gov/for_researchers/vinci/cdw.cfm

[16] Fihn SD, Francis J, Clancy C, Insights from advanced analytics at the Veterans Health Administration. Health Aff (Millwood). 2014;33(7):1203-1211. doi:10.1377/hlthaff.2014.0054 25006147

[17] D’Amico AV, Whittington R, Malkowicz SB, Biochemical outcome after radical prostatectomy, external beam radiation therapy, or interstitial radiation therapy for clinically localized prostate cancer. JAMA. 1998;280(11):969-974. doi:10.1001/jama.280.11.969 9749478

[18] Saha S, Freeman M, Toure J, Tippens KM, Weeks C, Ibrahim S Racial and ethnic disparities in the VA health care system: a systematic review. J Gen Intern Med. 2008;23(5):654-671. doi:10.1007/s11606-008-0521-4 18301951 PMC2324157

[19] US Department of Veterans Affairs VA priority groups. Published October 15, 2019 Accessed November 15, 2019. https://www.va.gov/health-care/eligibility/priority-groups

[20] Elixhauser A, Steiner C, Harris DR, Coffey RM Comorbidity measures for use with administrative data. Med Care. 1998;36(1):8-27. doi:10.1097/00005650-199801000-00004 9431328

[21] Chu Y-T, Ng Y-Y, Wu S-C Comparison of different comorbidity measures for use with administrative data in predicting short- and long-term mortality. BMC Health Serv Res. 2010;10:140. doi:10.1186/1472-6963-10-140 20507593 PMC2897792

[22] Kind AJH, Buckingham WR Making neighborhood-disadvantage metrics accessible—the Neighborhood Atlas. N Engl J Med. 2018;378(26):2456-2458. doi:10.1056/NEJMp1802313 29949490 PMC6051533

[23] Hu J, Kind AJH, Nerenz D Area deprivation index predicts readmission risk at an urban teaching hospital. Am J Med Qual. 2018;33(5):493-501. doi:10.1177/1062860617753063 29357679 PMC6027592

[24] University of Wisconsin School of Medicine Public Health About the Neighborhood Atlas. Accessed June 25, 2020. https://www.neighborhoodatlas.medicine.wisc.edu/

[25] Butler SS, Mahal BA, Lamba N, Use and early mortality outcomes of active surveillance in patients with intermediate-risk prostate cancer. Cancer. 2019;125(18):3164-3171. doi:10.1002/cncr.32202 31150125

[26] Loeb S, Walter D, Curnyn C, Gold HT, Lepor H, Makarov DV How active is active surveillance? intensity of followup during active surveillance for prostate cancer in the United States. J Urol. 2016;196(3):721-726. doi:10.1016/j.juro.2016.02.2963 26946161 PMC5010531

[27] Johansson J-E, Andrén O, Andersson S-O, Natural history of early, localized prostate cancer. JAMA. 2004;291(22):2713-2719. doi:10.1001/jama.291.22.2713 15187052

[28] Popiolek M, Rider JR, Andrén O, Natural history of early, localized prostate cancer: a final report from three decades of follow-up. Eur Urol. 2013;63(3):428-435. doi:10.1016/j.eururo.2012.10.002 23084329

[29] US Department of Veterans Affairs Minority veterans report. Published March 2017 Accessed August 26, 2020. https://www.va.gov/vetdata/docs/SpecialReports/Minority_Veterans_Report.pdf

[30] Ehdaie B, Carlsson S, Vickers A Racial disparities in low-risk prostate cancer. JAMA. 2019;321(17):1726-1727. doi:10.1001/jama.2019.2060 31063566

[31] Rebbeck TR Prostate cancer genetics: variation by race, ethnicity, and geography. Semin Radiat Oncol. 2017;27(1):3-10. doi:10.1016/j.semradonc.2016.08.002 27986209 PMC5175208

[32] Shiina M, Hashimoto Y, Kato T, Differential expression of miR-34b and androgen receptor pathway regulate prostate cancer aggressiveness between African-Americans and Caucasians. Oncotarget. 2017;8(5):8356-8368. doi:10.18632/oncotarget.14198 28039468 PMC5352406

[33] Haiman CA, Chen GK, Blot WJ, Characterizing genetic risk at known prostate cancer susceptibility loci in African Americans. PLoS Genet. 2011;7(5):e1001387. doi:10.1371/journal.pgen.1001387 21637779 PMC3102736

[34] Krishna S, Fan Y, Jarosek S, Adejoro O, Chamie K, Konety B Racial disparities in active surveillance for prostate cancer. J Urol. 2017;197(2):342-349. doi:10.1016/j.juro.2016.08.104 27596691

[35] Lu-Yao GL, Albertsen PC, Moore DF, Lin Y, DiPaola RS, Yao S-L Fifteen-year outcomes following conservative management among men aged 65 years or older with localized prostate cancer. Eur Urol. 2015;68(5):805-811. doi:10.1016/j.eururo.2015.03.021 25800944 PMC4575827

[36] Butler S, Muralidhar V, Chavez J, Active surveillance for low-risk prostate cancer in black patients. N Engl J Med. 2019;380(21):2070-2072. doi:10.1056/NEJMc1900333 31116925 PMC7374756

[37] Gurmankin AD, Polsky D, Volpp KG Accounting for apparent “reverse” racial disparities in Department of Veterans Affairs (VA)-based medical care: influence of out-of-VA care. Am J Public Health. 2004;94(12):2076-2078. doi:10.2105/AJPH.94.12.2076 15569955 PMC1448593

[38] Radomski TR, Huang Y, Park SY, Low-value prostate cancer screening among older men within the veterans health administration. J Am Geriatr Soc. 2019;67(9):1922-1927. doi:10.1111/jgs.16057 31276198 PMC7257436

[39] So C, Kirby KA, Mehta K, Medical center characteristics associated with PSA screening in elderly veterans with limited life expectancy. J Gen Intern Med. 2012;27(6):653-660. doi:10.1007/s11606-011-1945-9 22180196 PMC3358397

[40] Choyke PL, Loeb S Active surveillance of prostate cancer. Oncology (Williston Park). 2017;31(1):67-70. 28090626 PMC5555170

