# Supplementary material for: Comparison by Race of Conservative Management for Low-Risk and Intermediate-Risk Prostate Cancers in Veterans From 2004 to 2018
Source: JAMA Netw Open. 2020 Sep 28;3(9):e2018318. doi: 10.1001/jamanetworkopen.2020.18318 (PMC7522702; doi:10.1001/jamanetworkopen.2020.18318)
Supplement: Supplement. — eFigure 1. Cohort Selection eFigure 2. Observed Trends in Receipt of Conservative Management Among Veterans With Localized Prostate Cancer eFigure 3. Sensitivity Analysis of Time to Receipt of Definitive Therapy eTable 1. Code List eTable 2. Initial Management Strategy Over Time eTable 3. Univariable Log-Binomial Associations of Likelihood of Receipt of Conservative Management Across Patients With Low-Risk and Intermediate-Risk [file jamanetwopen-e2018318-s001.pdf]

Supplementary Online Content

Parikh RB, Robinson KW, Chhatre S, et al. Comparison by race of conservative management for low-risk and intermediate-risk prostate cancers in veterans from 2004 to 2018. *JAMA Netw Open*. 2020;3(9):e2018318. doi:10.1001/jamanetworkopen.2020.18318

eFigure 1. Cohort Selection

eFigure 2. Observed Trends in Receipt of Conservative Management Among Veterans With Localized Prostate Cancer

eFigure 3. Sensitivity Analysis of Time to Receipt of Definitive Therapy

eTable 1. Code List

eTable 2. Initial Management Strategy Over Time

eTable 3. Univariable Log-Binomial Associations of Likelihood of Receipt of Conservative Management Across Patients With Low-Risk and Intermediate-Risk

This supplementary material has been provided by the authors to give readers additional information about their work.

© 2020 Parikh RB et al. *JAMA Network Open.*

eFigure 1. Cohort Selection

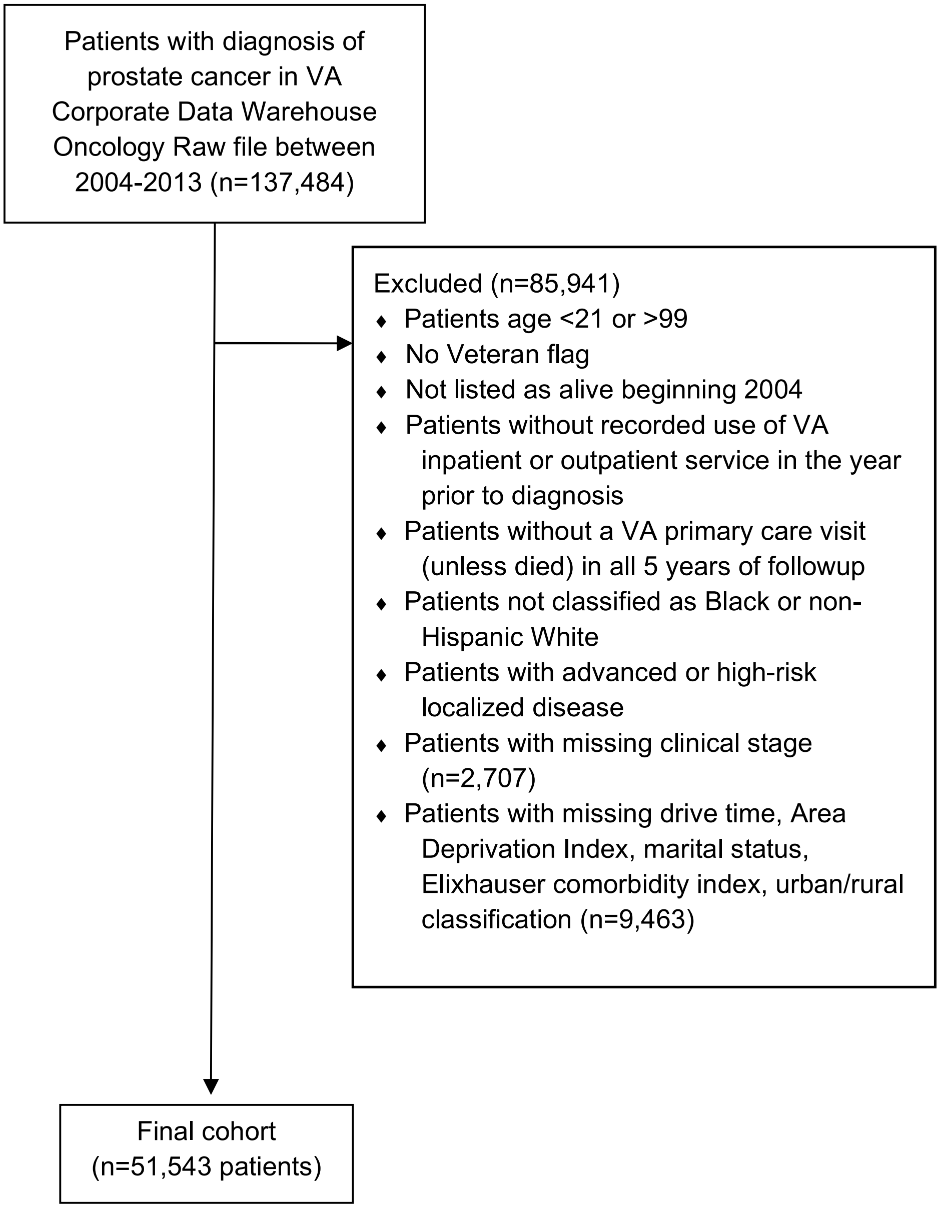

© 2020 Parikh RB et al. *JAMA Network Open.*

eFigure 2. Observed Trends in Receipt of Conservative Management Among Veterans With Localized Prostate Cancer

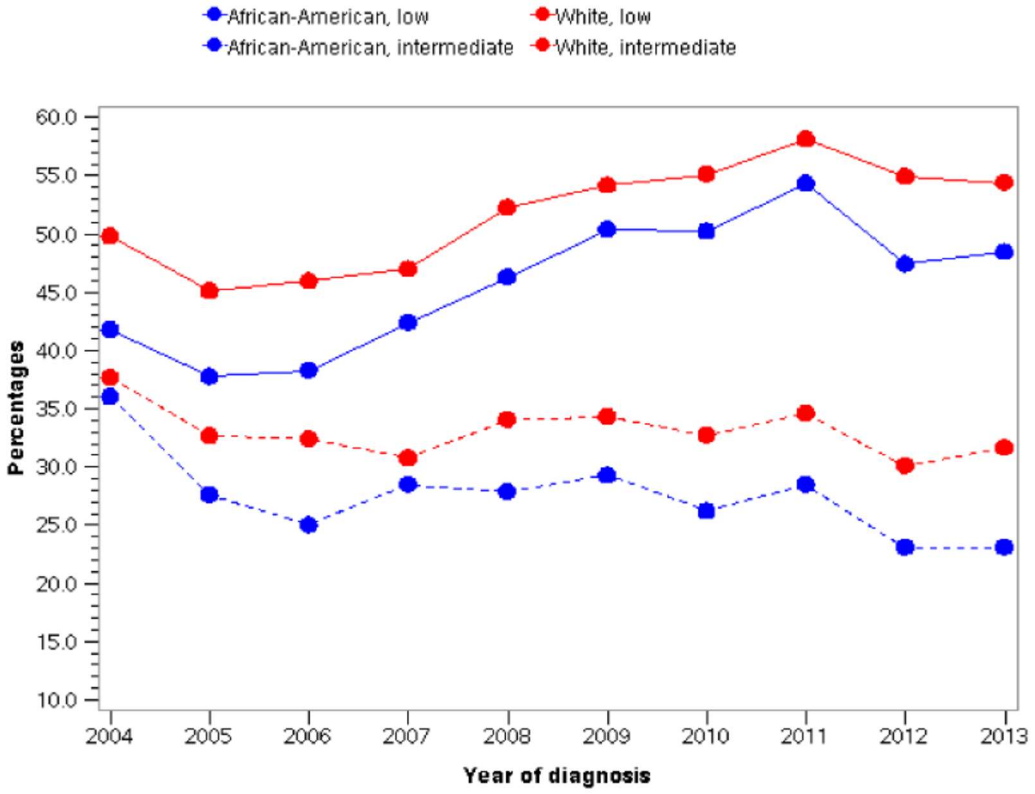

© 2020 Parikh RB et al. *JAMA Network Open.*

eFigure 3. Sensitivity Analysis of Time to Receipt of Definitive Therapy

eFigure 3A. Kaplan Meier curve of time to receipt of definitive therapy among African-American and White Veterans with low-risk localized prostate cancer who receive conservative management, 2004-2018, Sensitivity analysis

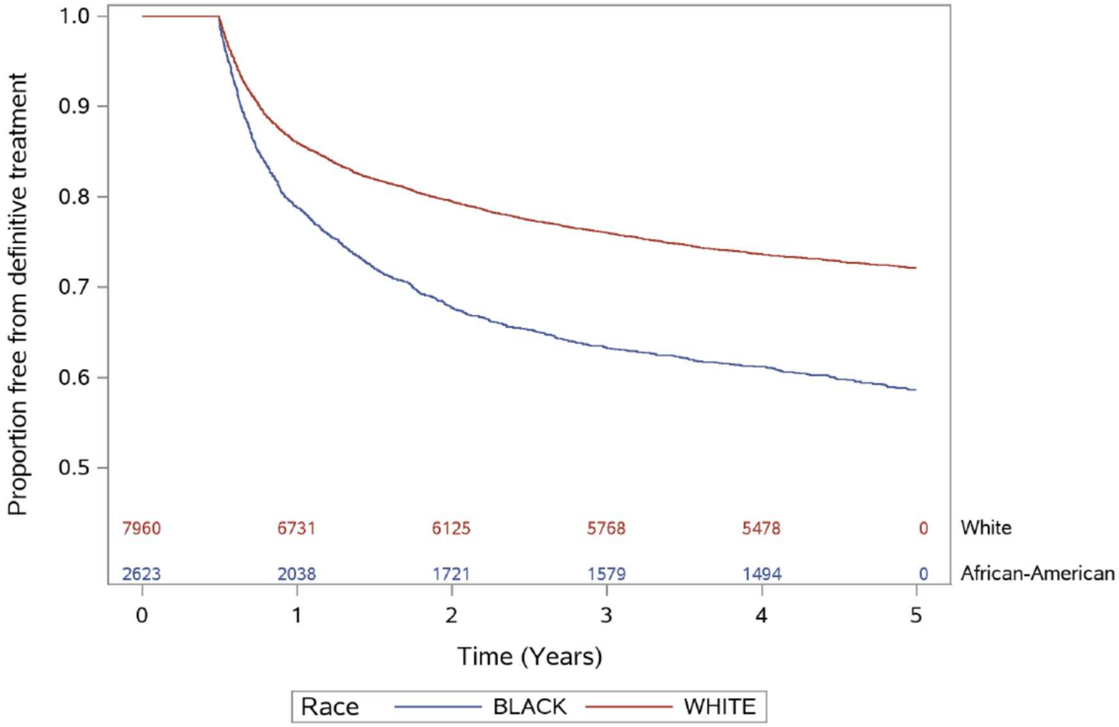

© 2020 Parikh RB et al. *JAMA Network Open.*

eFigure 3B. Kaplan Meier curve of time to receipt of definitive therapy among African-American and White Veterans with intermediate-risk localized prostate cancer who receive conservative management, 2004-2018, Sensitivity analysis

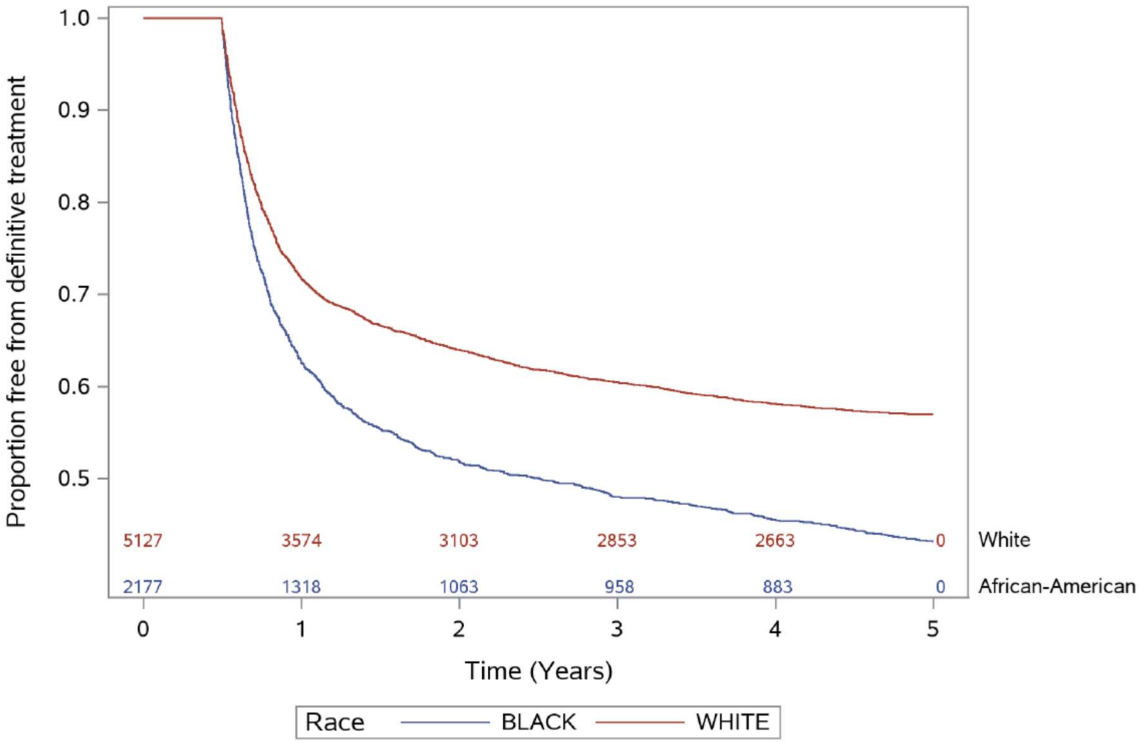

© 2020 Parikh RB et al. *JAMA Network Open.*

eTable 1. Code List

Variable	ICD-9 diagnosis/procedure code	CPT	HCPCS codes
Prostatectomy	60.5	55810-55815, 55821, 55831, 55840-55845, 55866	
Radiation	V58, V66.1, V67.1; 92.2-92.29	55869-55876, 77261-77431, 77499, 77522, 77720-77799	C1164-C11674, C1325, C1350, C1700-1806, C23632-C2643, G0174, G0256, G0261
Hormone Therapy	V07.59; 62.4x	54520, 54522, 54530, 54690	C9216, C9430, G03056, G9132, J0128, J1050, J1051, J1675, J1950, J3315, J9155, J9165, J9202, J9217-J9219, J9225-J9226, S0133, S0165, S0175, S9560

© 2020 Parikh RB et al. *JAMA Network Open.*

eTable 2. Initial Management Strategy Over Time

Intermediate risk (n = 27514)						
	African-American (n = 8526)			White (n = 18988)		
	Conservative management	Surgery	Radiation	Conservative management	Surgery	Radiation
2004-2007	721 (28.7%)	560 (22.3%)	993 (39.6%)	2073 (33.0%)	1375 (21.9%)	2220 (35.2%)
2008-2011	1046 (28.0%)	910 (24.3%)	1535 (41.0%)	2817 (33.9%)	1899 (22.9%)	3046 (36.7%)
2012-2013	525 (23.1%)	546 (24.0%)	1104 (48.5%)	1355 (30.1%)	1032 (23.5%)	1805 (41.1%)
Low risk (n = 24029)						
	African-American (n = 6304)			White (n = 17725)		
	Conservative management	Surgery	Radiation	Conservative management	Surgery	Radiation
2004-2007	923 (40.1%)	330 (14.3%)	946 (41.1%)	3228 (46.9%)	946 (13.7%)	2428 (35.3%)
2008-2011	1304 (50.3%)	297 (11.5%)	931 (35.9%)	4065 (54.9%)	829 (11.2%)	2346 (31.7%)
2012-2013	675 (47.9%)	118 (8.4%)	592 (42.0%)	1874 (54.7%)	298 (8.7%)	1216 (35.5%)

© 2020 Parikh RB et al. *JAMA Network Open.*

eTable 3. Univariable Log-Binomial Associations of Likelihood of Receipt of Conservative Management Across Patients With Low-Risk and Intermediate-Risk

Characteristic	RR (95% CI)	p-value
Age at diagnosis	1.015 (1.014-1.017)	<0.001
Absolute PSA	0.982 (0.980-0.986)	<0.001
Area Deprivation Index	0.999 (0.998-0.999)	<0.001
Driving time	1.011 (1.009-1.014)	<0.001
Elixhauser comorbidity index (ordinal)	1.249 (1.164-1.340)	<0.001
Urban/rural	1.063 (1.041-1.086)	<0.001
Race		
African-American	0.834 (0.814-0.855)	<0.001
White	1.0 [ref]	
Marital status		
Married	1.083 (1.060 -1.106)	<0.001
Single/Divorced	1.0 [ref]	

© 2020 Parikh RB et al. *JAMA Network Open.*
